# Spatiotemporal evolution and influencing factors of the allocation of social care resources for the older adults in China

**DOI:** 10.1186/s12939-023-02007-0

**Published:** 2023-10-18

**Authors:** Rong Peng, Jianhang Huang, Xueqin Deng

**Affiliations:** Institute of New Development, Guangdong University of Finance and Economics, Guangzhou, China

**Keywords:** Spatial distribution, Social elderly care resource, Dagum Gini Coefficient, Spatial panel regression models

## Abstract

**Background:**

The reasonable allocation of social care resources for the older adults is a key measure to actively respond to population aging. This study aims to evaluate the evolutionary trend, spatial differences and influencing factors of the social elderly care resources (SECR) allocation in China.

**Methods:**

This study constructed a comprehensive index system consisting of three dimensions: material resources, human resources and financial resources, to measure the level of SECR in mainland China. The Kernel density estimation was used to reveal the dynamic evolution trend, and Dagum Gini Coefficient and its decomposition method were used to investigate the equity of SECR allocation. Spatial panel regression models were used to analyze the influencing factors of the allocation of SECR.

**Results:**

The level of SECR is rising from 0.197 in 2013 to 0.208 in 2019. The middle-high- and high-level areas of SECR were mainly distributed in the eastern and western China. The Gini coefficient of SECR decreased from 0.262 in 2013 to 0.249 in 2019. Per capita GDP, the proportion of social welfare expenditure in GDP and the proportion of the tertiary industry in GDP have significant positive effects on the allocation of SECR. Population aging and the development of service industry exhibit significant negative spatial spillover effects on the allocation of SECR.

**Conclusions:**

The fairness of the allocation of SECR in China has been improved, while the spatial distribution is imbalanced. Economic development, fiscal input and the development of service industry have significant positive effects while population aging has significant negative effects on the SECR allocation.

**Supplementary Information:**

The online version contains supplementary material available at 10.1186/s12939-023-02007-0.

## Introduction

With the rapid population aging and family miniaturization, China’s traditional model of elderly care based on family informal care has gradually evolved into multi-social subject models including home-based care, institutional care and community-based care [15, 33, 49]. However, the shortage of social elderly care resources (SECR) and the imbalance between supply and demand has led to unfair and inefficient supply [22, 41] and unmet demand for elderly care services [6, 39]. The SECR is a general term for the physical elements of care services for older adults that rely on entities other than families, such as government, enterprises, communities, and non-profit organizations [29, 41]. The Chinese central government has been committed to improve SECR for the elderly people for the past several decades. For instance, the number of elderly care beds available for every 1,000 older adults increased from 21.48 in 2010 to 31.1 in 2020 [12]. However, there is a spatial mismatch between the diversity of facilities and the number of older adults [25, 50]. On the one hand, the high-quality and affordable institutions near the community where the elderly population gathers are hard to access because of the shortage of beds [46]. On the other hand, the vacancy rate of institutional care beds in China has reached to 50 percent [36], which indicates a huge waste of SECR.

To promote the efficient use of elderly care resources, it is important to optimize the allocation of SECR [20, 43]. With the rapid increasing demand for elderly care, the supply of social elderly care services is not only constrained by limited human, material and financial resources [43], but also by the spatial configuration of SECR [47]. The allocation of public welfare resources, such as SECR, in a region depends not only on its own economic condition and input, but also on the allocation of public welfare resources in other regions, particularly in its adjacent areas [25]. Due to the spatial correlation of SECR, a reasonable distribution of SECR in space may reduce the waste of resources caused by spatial mismatch. In China, the government plays an important guiding role in the allocation of public welfare resources [49]. It is necessary to study the spatial characteristics and influencing factors of SECR allocation, which can provide evidence-based support for the government to comprehensively understand the current situation and to identify pathways for optimizing resource allocation.

The existing literature contribute to evaluating the supply–demand matching [37], the suitability [19], regional disparities [47] and sustainability [28, 50] of the elderly care service facilities. Coupling coordination coefficient and deviation degree index method was used to explore the matching relationship between the elderly care resources and the elderly population in China [27, 51]. Due to the impact of population size, structure and distribution on the existing allocation of public care resources, there are some issues in SECR allocation, such as insufficient effective supply [15, 46], uneven resource allocation between regions [22, 25], and a mismatch between service facilities and the elderly population [9, 10]. Population aging, urbanization rate, family structure, government financial expenditure and government intervention capability are important factors that influence the SECR allocation [32, 53].

It is particularly noteworthy that the research on the spatial layout of elderly care service facilities has attracted wide attention from some interdisciplinary researchers in recent years [21]. These studies used spatial analysis tools to examine the spatial distribution equilibrium of elderly care facilities [25, 53] and spatial accessibility [5, 11, 8, 31]. The agent-based simulation was used to predict the demand and provision of elderly care facilities [7] and facilitate the evaluation of planning policies for elderly care services [45]. Tao et al. [48] used the particle swarm optimization algorithm to establish an optimization model for facility layout, aiming to improve the fairness of the elderly care facilities. The previous literature has elucidated the influence of spatial factors on the allocation of SECR, providing us with a valuable perspective to study the spatial allocation of SECR in China.

However, the existing studies have at least the following deficiencies. First, it overlooked the comprehensive measurement of SECR. Despite the rich connotation of SECR, most studies tend to focus on specific measurement indicators, such as the number of elder care institutions [53] and the number of beds in care institutions [48]. Such a measurement index has the deficiency of inadequate representativeness, and the measurement results are difficult to comprehensively reflect the overall level of resource allocation. Second, there are few literatures investigating the influencing factors of SECR allocation in China from a spatial perspective. Since there is a significant spatial correlation in the distribution of SECR [27], it will affect the objectivity of the results to examine the influencing factors of SECR allocation without considering the spatial structure. Third, existing studies often use cross-sectional or mixed data instead of panel data, which leads to the endogeneity in regression models, making it difficult to reveal causal relationships in the research findings.

This study aims to evaluate the evolutionary trend, spatial differences and influencing factors of SECR allocation in China. Based on the actual connotation of Chinese elderly care resources, this study constructs an evaluation index system for SECR and calculates the level of SECR using the panel data from 31 Chinese provincial administrative regions. The spatial statistics method is used to explore spatial correlation and investigate the influencing factors of SECR allocation.

The marginal contribution of this study includes at least three aspects. Firstly, this study constructs a measurement indicator system for SECR from multiple dimensions to provide a more comprehensive assessment of the development level and equity of SECR allocation in China, the largest emerging economy in the world. Secondly, this study provides an academic understanding of the spatial evolutionary trends and equity of SECR. Thirdly, this research endeavors to explore the influencing factors of SECR allocation from the perspective of spatial effects, providing evidence-based support for the government to formulate policies that promote the allocation of elderly care resource.

## Index system for measuring the level of SECR

There is no uniform index system of elder care resources in the literature. According to the core connotation of resources, the most basic elements are material resources, human resources and financial resources [27, 51]. This study constructs a quantitative indicator system to measure the level of SECR, which consists of material resources, human resources and financial resources provided by the subjects outside the family for the elderly.

Material resources serve as the carrier and location for the provision of elderly care services. The elderly care institutions, community-based care institutions, medical and health institutions, geriatric hospitals are the main venues for providing social elder care services in China. Elderly care resources of these institutions include the material facilities and human resources. Common indicators used to measure material resources include the number of institutions and the number of beds in these institutions [43, 48, 53].

Human resources are an important guarantee for providing care services, including staff and nurses in social elderly care service institutions [3, 40]. In addition, social workers are also crucial personnel to improve the level of social public services. According to the Chinese guideline to promote development of national undertakings for the aged and improve the elderly care service system during the 14th Five-Year Plan period (2021–2025), it was clearly pointed out that by 2025, the targeted value of the number of social workers per 1,000 elderly people should be more than 1 person.

Financial resources refer to the social financial support for the older adults to access social care services. It is a key factor that influences the development of social elder care services [7, 27, 38]. In China, basic pension insurance and social medical insurance provide financial support for the living expenses and medical expenses of the older persons, respectively. The aging subsidies issued by central or local governments for older people who meet certain conditions are unique additional source of funding. The government spending on social public health directly affects the supply of social elder care services [29, 49].

Based on the aforementioned connotation of SECR and the availability of data, this study constructs an evaluation index system from the three dimensions of material resources, human resources and financial resources, including 3 first-level indicators and 17 s-level indicators (as shown in Table 1). The material resources include six indicators: the number of institutional care beds per thousand older adults, the number of elderly care institutions per thousand older adults, the number of care beds in communities per thousand older adults, the number of community care institutions per thousand older adults, the number of beds in medical and health institutions per thousand older adults, the number of beds in geriatric hospital per thousand older adults. The human resource includes five indicators: the number of employees in elderly care institutions per thousand older adults, the number of employees in community-based care institutions per thousand older adults, the number of health technicians per ten thousand people, the proportion of registered nurses in health technicians, the number of social workers per thousand older adults. The financial resources include six indicators: social pension insurance expenditure per capita, social medical insurance expenditure per capita, public health expenditure per capita, the proportion of older adults receiving old-age subsidies, the proportion of older adults receiving elder care subsidies, the proportion of older adults receiving pension subsidies. Due to the unsuitability of aggregate index for direct comparison, each index is expressed in the forms of average index or a proportional index.

**Table 1:** Index system for evaluating the level of social elderly care resources

Dimensions	Indicators	Calculation formula	Unit	Weights*
Material resources (24.37%)	Number of institutional care beds per thousand older adults	Number of institutional care beds/population of older adults*1000	Beds/1000 people	3.34%
	Number of elder care institutions per thousand older adults	Number of elderly care institutions/population of older adults*1000	Institutions/1000 people	3.26%
	Number of care beds in communities per thousand older adults	Number of care beds in communities/population of older adults*1000	Beds/1000 people	5.40%
	Number of community-based care institutions per thousand older adults	Number of community-based care institutions/population of older adults*1000	Institutions/1000 people	4.95%
	Number of beds in medical and health institutions per thousand older adults	Number of beds in medical and health institutions/population of older adults*1000	Beds/1000 people	4.68%
	Number of beds in geriatric hospital per thousand older adults	Number of beds in geriatric hospitals/population of older adults*1000	Beds/1000 people	2.73%
Human resources (25.15%)	Number of employees in elderly care institutions per thousand older adults	Number of employees in elderly care institutions/population of older adults*1000	Employees/1000 people	5.08%
	Number of employees in community-based care institutions per thousand older adults	Number of employees in community-based care institutions/population of older adults*1000	Employees/1000 people	6.14%
	Number of health technicians per ten thousand people	Number of health technicians/total population*10,000	Technicians/10000 people	0.90%
	Proportion of registered nurses in health technicians	Number of registered nurses/number of health technicians*100	%	3.25%
	Number of social workers per thousand older adults	Number of social workers/population of older adults*1000	Employees/1000 people	9.79%
Financial resources (50.48%)	Social pension insurance expenditure per capita	Basic pension insurance expenditure/number of retired employees	Yuan/person	8.87%
	Social medical insurance expenditure per capita	Basic medical insurance expenditure/insured population	Yuan/person	7.14%
	Public health expenditure per capita	(Government expenditure on health + social expenditure on health)/total population	Yuan/person	4.60%
	Proportion of older adults receiving old-age subsidies	Number of older adults receiving old-age subsidies/population of older adults*100	%	6.67%
	Proportion of older adults receiving elderly care subsidies	Number of older adults receiving elderly care subsidies/population of older adults*100	%	10.67%
	Proportion of older adults receiving pension subsidies	Number of older adults receiving pension subsidies/population of older adults*100	%	12.55%

^*^the weight is the average of the weights over the years calculated by the entropy weight method

## Methods

### Data sources

The data are from the 2014–2020 China Civil Affairs Statistics Yearbook, China Statistics Yearbook, China Health Statistics Yearbook, and China Cultural Relics Statistics Yearbook. The data obtained in this study cover 31 provincial-level administrative regions in mainland China. The time span of the data is from 2013 to 2019. Microsoft Excel 2016 and Stata 17 were used for data processing and analysis. ArcGis 10.2 was used for generating maps.

### Entropy weight method for calculating the comprehensive index of SECR

The performance or development level of SECR is calculated by using the entropy weight method. The entropy weighting method is widely used to evaluate comprehensive development level [16, 24, 38]. The information entropy is used in this method to reflect the amount of information obtained for weighting [42]. The information entropy can fully reflect all the information in the sample, and its results have high reliability and strong adaptability. The entropy weighting method is an objective weighting method and more liable than the subjective method of comprehensive evaluation of multiple indicators since it avoids the interference of subjective factors [16]. In this study, we repeat the following steps to calculate the development level of SECR for any fixed year from 2013 to 2019.

Firstly, the raw data ${Y}_{ij}$ (The subscript i refers to the indicator i (i = 1,2,⋯,17) and j refers to indicator province j ($j=\mathrm{1,2}, \cdots , 31$)) was standardized using the following equation in order to get rid of the influence of dimension and magnitude. For positive values, ${Y}_{ij}^{\mathrm{^{\prime}}}=\frac{{Y}_{ij}-min{Y}_{ij}}{max{Y}_{ij}-min{Y}_{ij}}$; for negative values, ${Y}_{ij}^{\mathrm{^{\prime}}}=\frac{{maxY}_{ij}-{Y}_{ij}}{max{Y}_{ij}-min{Y}_{ij}}$.

Secondly, the information entropy ${E}_{i}$ and the weight ${W}_{i}$ for the indicator i were calculated by the following formulas:

$${E}_{i}=-k\sum_{j}^{31}{f}_{ij}\mathrm{ln}\left({f}_{ij}\right) (where, {f}_{ij}=\frac{{Y}_{ij}^{\mathrm{^{\prime}}}}{\sum_{j=1}^{31}{Y}_{ij}^{\mathrm{^{\prime}}}};k=\frac{1}{ln31})$$, if $${f}_{ij}=0$$, $${f}_{ij}\mathrm{ln}\left({f}_{ij}\right)=0$$.

$${W}_{i}=\frac{1-{E}_{i}}{17-\sum_{i=1}^{17}{E}_{i}}$$

Thirdly, the composite development index of SECR in province j was calculated as follows:

$${Z}_{j}=\sum_{\mathrm{i}}{\mathrm{W}}_{\mathrm{i}}{Y}_{ij}^{\mathrm{^{\prime}}}$$

### The Kernel density estimation method

This study uses the Kernel density estimation method to examine the dynamic evolution trend of SECR in China from 2013 to 2019. The Kernel density estimation method uses continuous density curves to describe the distribution characteristics of variables [56], and it is currently widely used in the study of spatial disequilibrium distribution [30, 34]. By observing the Kernel density curve, information such as the distribution position, peak characteristics, distribution ductility, and polarization trend of variables can be obtained. The distribution position can reflect the allocation level of regional SECR,the height of the peak reflects the size of the gap in the allocation level of SECR, and the number of peaks reflects the polarization degree of the allocation level of SECR,the distribution ductility can reflect the differences between the highest level and the lowest level of elder care resource allocation.

### Dagum Gini Coefficient and its decomposition method

This study uses the Dagum Gini coefficient and its decomposition method to measure and analyze the differences in the allocation level of SECR. This method overcomes the limitations of the traditional Gini coefficient and Theil index, enabling effective analysis of the causes of regional differences, resolving the problem of overlap between subgroups, and achieving precise decomposition of the net gap contributions between regions to the overall regional gap [14]. With reference to Dagum [14], the calculation formula of Dagum’s Gini coefficient is

$$\mathrm{G}={\sum }_{j=1}^{k}{\sum }_{h=1}^{k}{\sum }_{i=1}^{{n}_{j}}{\sum }_{r=1}^{{n}_{h}}\left|{y}_{ji}- {y}_{hr}\right| / {2n}^{2}\overline{y }$$

where, n is the number of Chinese provincial administrative regions (*n* = 31 in this study), k is the number of regional divisions (k = 3 in this study, denoting Western China, Central China and Eastern China), ${n}_{j}$ and ${n}_{h}$ represents the number of provinces contained in regions j and h respectively. $\overline{y }$ is the average value of SECR of all regions, ${y}_{ji}$ represents the SECR level of province i in region j, ${y}_{hr}$ represents the SECR level of province r in region h.

According to Dagum [14], the Dagum’s Gini coefficient can be divided into three components.

$$\mathrm{G}={\mathrm{G}}_{w}+{\mathrm{G}}_{nb}+{\mathrm{G}}_{i}$$

where, ${G}_{w}$ is the inter-regional variance contribution, ${G}_{nb}$ is the intra-regional variance contributions, ${G}_{i}$ is the super-variable density contribution. Their detailed calculation formulas are given in the literature [14].

### Spatial analysis method

Firstly, in order to reveal the spatial characteristic of SECR allocation in China, the comprehensive SECR index in 2013, 2015, 2017 and 2019 was visualized using ArcGIS software. The Natural Breaks (Jenks) was used to classify 31 provincial-level administrative regions into four categories: Low-level area, medium–low-level area, medium–high-level area and high-level area. The Natural Breaks method (Jenks) can group the similar values most appropriately to ensure significant differences between groups and small differences within groups [26].

Secondly, spatial autocorrelation test is used to verify whether there is spatial correlation in the SECR allocation. The global Moran index (Moran’s I) is usually used to test the spatial autocorrelation [2, 34, 38]. Moran’s I is calculated as follows:

$$\mathrm{Moran{\prime}}\mathrm{s I}=\frac{\mathrm{N}}{{\mathrm{S}}_{0}}\frac{{\sum }_{\mathrm{i}=1}^{\mathrm{N}}\sum_{\mathrm{j}=1}^{\mathrm{N}}{\mathrm{w}}_{\mathrm{ij}}({\mathrm{y}}_{\mathrm{i}}-\overline{\mathrm{y}})({\mathrm{y}}_{\mathrm{j}}-\overline{\mathrm{y}})}{{\sum }_{\mathrm{i}=1}^{\mathrm{N}}{({\mathrm{y}}_{\mathrm{i}}-\overline{\mathrm{y}})}^{2}}$$

where $\mathrm{N}$ denotes the number of space elements, ${y}_{i}$ and ${y}_{j}$ represent the comprehensive index $\mathrm{y}$ in space units i and j, $\overline{y}$ denotes the mean value of the variable y. ${\mathrm{w}}_{\mathrm{ij}}$ represents the elements in the spatial weight matrix. In this study, it was defined as a binary adjacency matrix with the elements equal to 1 or 0. If i and j are different, ${\mathrm{w}}_{\mathrm{ij}}=1$, otherwise ${\mathrm{w}}_{\mathrm{ij}}=0$. ${S}_{0}$ is the sum of all elements of the space weight matrix.

The Global Moran’s I is within the range of [-1, 1]. Positive values indicate positive spatial autocorrelation, negative values indicate negative spatial autocorrelation, and 0 indicate spatial randomness. The larger the absolute value, the stronger the spatial correlation. When the results of spatial autocorrelation tests show the presence of spatial dependence among the observed subjects, it is necessary to establish a spatial measurement model to reflect spatial effects [2].

Thirdly, the spatial panel models were built to investigate the influencing factors of SECR allocation. The dependent variable is the comprehensive index of SECR. According to the literature, the SECR allocation is affected by economic development, fiscal input, service industry development, population aging and natural environment [32, 53]. Based on data availability, six indicators were selected as independent variables. The GDP per capita represents economic factors [53, 54]. The proportion of social welfare expenditure in GDP represents the fiscal input for the older adults [53]. The proportion of the tertiary sector represents the level of the service industry development [38]. The proportion of population aged 65 + and the old-age dependency ratio represent the factors related to population aging [34, 53]. The per capita park and green space area represent the environmental factor [50]. The statistical description of the dependent and independent variables is shown in Table 2. The spatial error model (SEM), the spatial autoregressive (SAR) model, and spatial Dubin model (SDM) are used to test the spatial spillover effect. The models of SEM (Model 1), SAR (Model 2) and SDM (Model 3) in this study can be expressed as follows:

Model 1 $${y}_{it}=\beta {x}_{it}+\lambda W{\mu }_{it}+{\varepsilon }_{it}$$

Model 2 $${y}_{it}=\rho W{y}_{it}+\beta {x}_{it}+{\varepsilon }_{it}$$

Model 3 $${y}_{it}=\rho W{y}_{it}+\beta {x}_{it}+\theta W{x}_{it}+{\varepsilon }_{it}$$

where, ${y}_{it}$ represents the level of elder care resources in province i in year t, ${x}_{ij}$ represents the vector of independent variable. $\lambda$, $\rho$ and $\theta$ are spatial autoregression parameters. $\beta$ is the regression parameter. $W$ is the binary adjacency matrix. Both $W{y}_{it}$ and $W{x}_{it}$ are spatially lagged variables. $W{u}_{it}$ is a spatially lagged error term. ${\varepsilon }_{it}$ is an error term.

**Table 2:** The statistical description of the dependent and independent variables

Variables	Observed value	Mean	Std.Dev	Min	Max
Ln (the comprehensive index of SECR)	217	-1.635	0.458	-2.790	-0.295
Ln (the per capita GDP)	217	1.661	0.411	0.839	2.799
Ln (the proportion of social welfare expenditure in GDP)	217	-2.301	0.498	-3.342	-0.540
Ln (the proportion of the tertiary industry in GDP)	217	-0.744	0.174	-1.139	-0.180
Ln (the proportion of the older adults aged 65 +)	217	-2.167	0.427	-2.754	-0.195
Ln (the old age dependency ratio)	217	-1.965	0.240	-2.658	-1.435
Ln (the park green space area per capita)	217	2.555	0.216	1.766	3.047

*Ln* natural logarithm; SECR, social elderly care resources

The LM test, LR test and Wald test were used to make comparison among SDM, SEM and SAR models in order to select the most appropriate model. The Hausman’s test was used to determine fixed effect or random effect. In order to test the stability of the spatial model, we also ran a SDM model based on the geographical distance matrix.

## Results

### Evolutionary trend of elderly care resource allocation

**Fig. 1:**
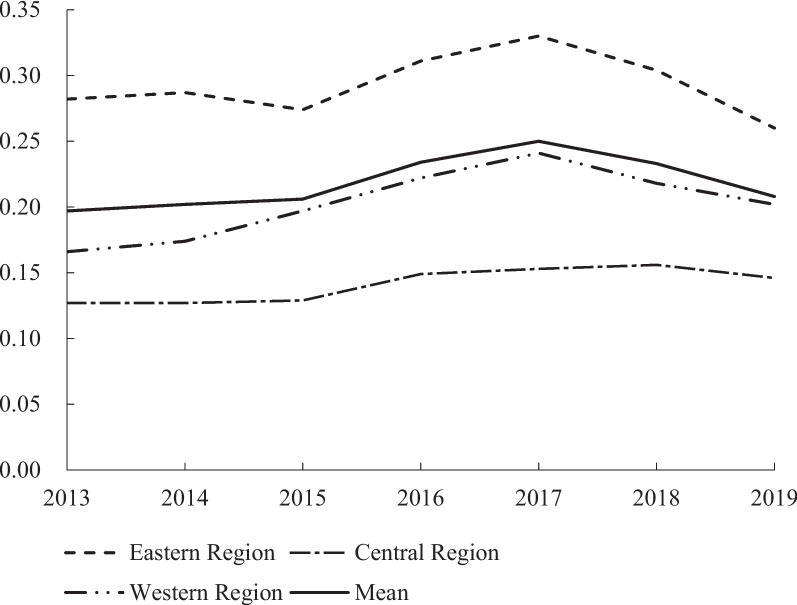
The comprehensive index of the SECR in China (2013–2019)

Figure 1 is a time trend chart of comprehensive index of SECR in China from 2013 to 2019. It can be seen that the average value of the comprehensive index of China’s SECR presents an inverted "V" shape, rising from 0.197 in 2013 to 0.250 in 2017, and then declining to 0.208 in 2019. Overall, there is an upward trend (see the Supplementary Table S1 for the values of the comprehensive index). The SECR index is highest in eastern China (0.293), followed by western China (0.203) and central China (0.141). Among the three dimensions of SECR, the weight of financial resources is the largest (50.48%) (see Table 1), indicating its largest contribution to the comprehensive evaluation system for SECR allocation.

Table 3 shows the classification range of SECR levels from 2013to 2019 using the Natural Breaks Classification (Jenks) in ArcGIS software. It shows that the maximum values for each internal in the years 2013–2019 exhibit a trend of initially rising and then declining, which is consistent with the results of Fig. 1 and Table S1 (See the supplementary file), indicating that the SECR level experienced a process of initial increase followed by a decrease.

**Table 3:** Ranges of the social elderly care resource level classification

Year	Low level area	Medium–low level area	Medium–high level area	High level area
2013	0.082–0.130	0.130–0.198	0.198–0.288	0.288–0.615
2014	0.061–0.162	0.162–0.239	0.239–0.340	0.340–0.587
2015	0.075–0.141	0.141–0.225	0.225–0.363	0.363–0.586
2016	0.092–0.170	0.170–0.247	0.247–0.356	0.356–0.695
2017	0.103–0.147	0.147–0.243	0.243–0.384	0.384–0.744
2018	0.105–0.188	0.188–0.261	0.261–0.382	0.382–0.664
2019	0.115–0.153	0.153–0.214	0.214–0.402	0.402–0.578

**Fig. 2:**
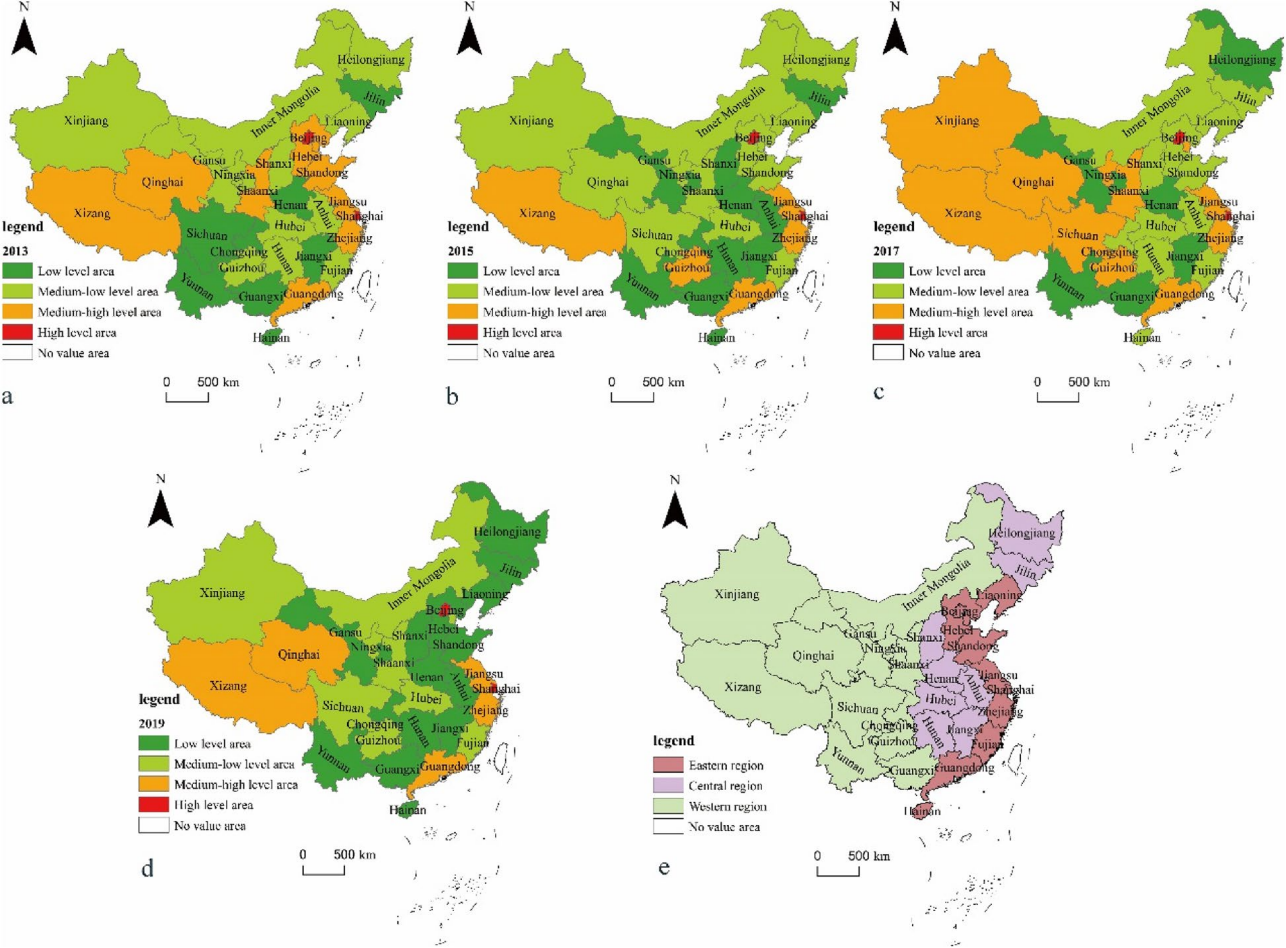
The spatial distribution of China’s SECR in 2013, 2015, 2017 and 2019

Figure 2 shows the spatial distribution of China’s SECR in 2013 (sub-figure a), 2015 (sub-figure b), 2017 (sub-figure c), and 2019 (sub-figure d). It can be observed that in each year, most provincial administrative regions belong to the low-level areas or medium–low-level areas, indicating a generally low overall level of SECR in China; Provinces with a medium–high or high-level- SECR are mainly distributed in the western and eastern regions of China (See sub-figure e for location of Western China, Central China and Eastern China). The spatial distribution of China’s SECR is imbalanced, with high-level areas located on the periphery and low-level areas in the middle. In areas with a low-level SECR, the surrounding areas also tend to have low SECR level. From 2013 to 2019, the grade level of SECR in most provinces remained relatively stable.

**Fig. 3:**
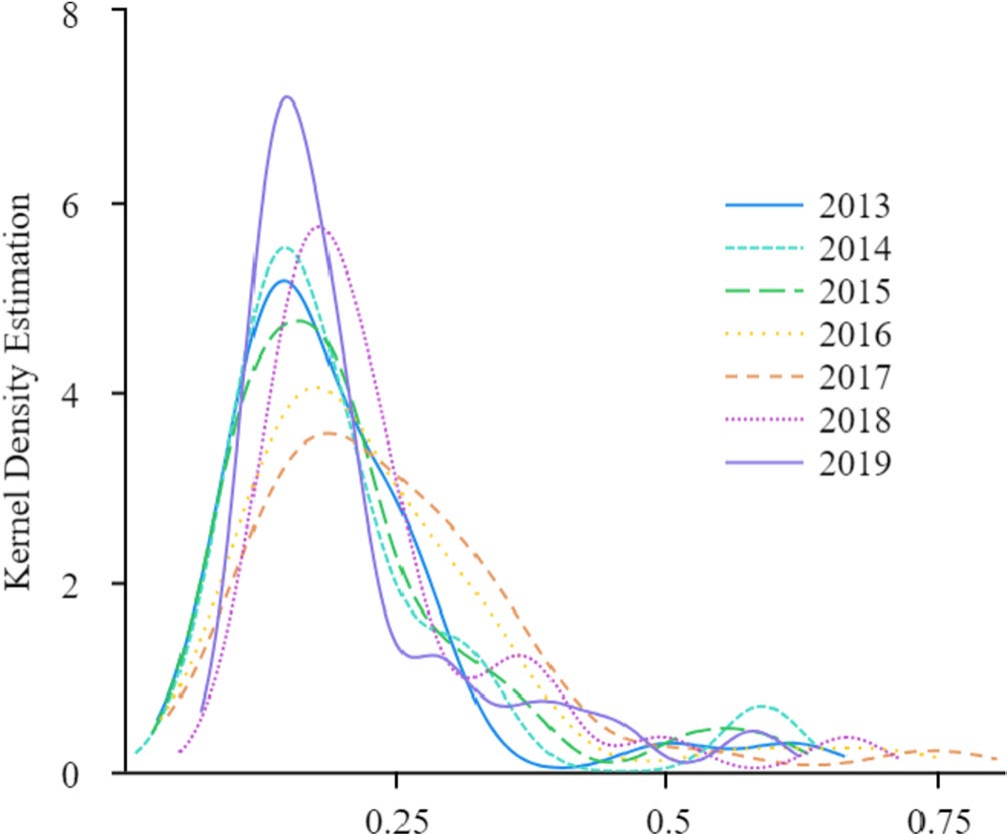
The nuclear density estimation of the SECR allocation in China (2013–2019)

Figure 3 illustrates the dynamic evolution trend of SECR allocation in China from 2013 to 2019. The center of the Kernel density estimation function continued to move to the right during the period from 2013 to 2017. However, from 2017 to 2019, it slightly moved to the left but did not exceed the level of 2013. This indicates that from 2013 to 2019, China's SECR allocation evolved from low-level to high-level, but with a sight regression. The main peak of SECR level experienced fluctuations followed by an upward trend, while the width of the curve slightly narrowed. This indicates that the dispersion of SECR in China between 2013 and 2019 decreased, and the absolute differences between regions decreased as well. The extension of the Kernal density curve indicates a noticeable right-tail swing in the levels of SECR, with a trend of widening first and then narrowing. This indicates that the gap between the regions with SECR levels and those with low SECR levels was narrowed during the observation period.

### The equity of SECR allocation

Table 4 shows the trend of Dagum Gini coefficient for SECR allocation in China and its eastern, central, and western regions. From the overall national perspective, the Gini coefficients shows a declining trend from 2013 to 2019. In 2014, the Gini coefficient reached its highest value of 0.284. In 2018, the Gini coefficient reached its lowest value of 0.242, indicating a slow increase in fairness of SECR in China. The eastern region has the highest Gini coefficient, followed by the western and central regions. Taking 2019 as an example, the Gini coefficient for the eastern region was 0.298, higher than the Gini coefficient of 0.189 for the western region and 0.068 for the central region. Looking internally within each region, the eastern region showed a clear upward trend in the Gini coefficient, increasing from 0.254 in 2013 to 0.298 in 2019, consistently at a higher level. The Gini coefficient for the western region had a relatively stable time series (range = 0.074), consistently at around 0.2; the Gini coefficient for the central region decreased from 0.105 in 2013 to 0.068 in 2019 and remained at a relatively low level throughout.

**Table 4:** The Dagum Gini coefficient and its differential decomposition

		The Dagum Gini coefficient						Contribution rate (%)		
Year	Total	Central Region	Eastern Region	Western Region	Eastern & Central Region	Eastern & Western Region	Central & Western Region	Within group	Between group	Super-variable density
2013	0.262	0.105	0.254	0.149	0.386	0.298	0.177	26.382	66.048	7.570
2014	0.284	0.099	0.286	0.196	0.391	0.314	0.213	28.380	60.600	11.020
2015	0.281	0.170	0.270	0.223	0.379	0.282	0.263	29.836	52.798	17.366
2016	0.26	0.119	0.278	0.176	0.362	0.266	0.229	29.317	56.325	14.358
2017	0.259	0.122	0.259	0.170	0.369	0.255	0.261	28.046	57.405	14.549
2018	0.242	0.106	0.269	0.152	0.333	0.260	0.193	29.120	56.220	14.661
2019	0.249	0.068	0.298	0.189	0.313	0.276	0.185	31.156	46.204	22.640

Looking at the Gini coefficient for the three regions, the highest Gini coefficient is observed in the central and eastern regions, followed by the eastern and western regions, and then the central and western regions. This indicates that the largest gap exists between the eastern and the central regions, followed by the gap between the eastern and central regions, and the central and western regions. The Gini coefficient for the eastern-central region in China decreased from 0.386 in 2013 to 0.313 in 2019, representing a decrease by 18.912%. The time series of the Gini coefficient of eastern-western and central-western regions of China were relatively stable with little variations.

In terms of contribution rate of regional gap to SECR, the inter-regional gap had the highest contribution rate, followed by the intra-regional gap and super-variable density. The contribution rate of inter-regional gap had decreased significantly from 66.048% in 2013 to 46.204% in 2019, representing a decline of 30.045%. The contribution rate of intra-regional gap had slightly increased, fluctuating between 26 and 32%. The contribution rate of super-variable density shows a clear upward trend, rising from 7.570% in 2013 to 22.640% in 2019, indicating an enhancement in the overlapping phenomenon among regions.

### Spatial autocorrelation test results

Table 5 presents the results of the spatial autocorrelation test. The Moran’s I values are significantly positive at the 0.05 level of significance except 2015 and 2017. This indicates that there is a certain degree of spatial autocorrelation in the SECR, and the phenomenon of spatial agglomeration does exist [13, 54]. Therefore, it is appropriate to use a spatial econometric model to empirically analyze the spatial effects.

**Table 5:** Moran’s I index of the social elderly care resource (2013–2019)

Year	2013	2014	2015	2016	2017	2018	2019
Global Moran’s value	0.222	0.156	0.08	0.107	0.065	0.127	0.105
P-value	0.026**	0.046**	0.14	0.096*	0.17	0.081*	0.096*

^*^, and ** indicate significance at the level of 0.05 and 0.01 respectively

Table 6 displays the results of tests related to model selection. The robust LM-error test and robust LM-lag test are both significant, indicating that both SEM and SAR are appropriate. The LR test and Wald test reject the null hypothesis that SDM can be simplified to SEM or SAR, indicating that SDM performs better than SEM and SAR. The Hausman’s test rejects the null hypothesis at significance level of 5%, indicating the selection of the fixed effect model.

**Table 6:** The test results related to model selection

		Statistics	*P*-value
LM test	SEM	2.174	0.140
	Robust SEM	7.040 **	0.008
	SAR	9.066 **	0.003
	Robust SAR	13.932 ***	0.000
LR test	SDM can be simplified to SAR	15.550 **	0.004
	SDM can be simplified to SEM	20.390 ***	0.000
Wald test	SDM can be simplified to SAR	13.460 *	0.019
	SDM can be simplified to SEM	14.530 *	0.013
Hausman test	SAR	33.170 ***	0.000
	SEM	28.430 ***	0.000
	SDM	27.470 *	0.011

^*^, **, and *** indicate significance at the level of 0.05, 0.01 and 0.001 respectively

### Spatial panel model results

Table 7 shows the results of the ordinary panel model (OLS) and four spatial panel models. It can be seen that, except for the variable “per capita park green space area,” the significance of the regression coefficients is consistent in the SDM model based on binary adjacency matrix (SDM1) and the SDM model based on geographical distance matrix (SDM2). The results of OLS, SAR and SEM models are consistent with those of SDM1 model, indicating that the SDM1 model performs better than SDM2. Local economy, fiscal input and service industry development can significantly contribute to the SECR level. In the SDM1 model, the spatial autoregressive coefficient is -0.402 and it passed the significance test at a 0.01 level, indicating a significant impact of adjacent areas' SECR on the observed region. Per capita GDP (coefficient = 0.516, *P*-value = 0.001), social welfare expenditure as a percentage of GDP (coefficient = 0.155, *P*-value = 0.043), and the proportion of the tertiary industry (coefficient = 0.797, *P*-value = 0.016) has a significantly positive impact on the allocation of SECR. The proportion of the older adults over 65 years old (coefficient = -0.287, *P*-value = 0.017) and the old age dependency ratio (coefficient = -0.616, *P*-value = 0.000) are both significantly negatively correlated to the SECR level, indicating that higher aging levels correspond to lower SECR level.

**Table 7:** Estimated coefficients of the spatial SEM, SAR, SDM models

Variables	OLS	SAR	SEM	SDM1	SDM2
Rho		-0.237***		-0.402***	-1.487***
		(0.091)		(0.074)	(0.252)
Lambda			-0.372***		
			(0.099)		
Ln (the per capita GDP)	0.610***	0.387**	0.369**	0.516***	0.408**
	(0.069)	(0.171)	(0.159)	(0.177)	(0.160)
Ln (the proportion of social welfare expenditure in GDP)	0.225***	0.103	0.138	0.155**	0.177**
	(0.058)	(0.086)	(0.091)	(0.079)	(0.088)
Ln (the proportion of the tertiary industry in GDP)	0.642***	0.802***	0.720***	0.797***	0.554**
	(0.163)	(0.265)	(0.253)	(0.235)	(0.231)
Ln (the proportion of the older adults aged 65 +)	0.0309	-0.227	-0.300*	-0.287*	-0.429***
	(0.076)	(0.166)	(0.172)	(0.149)	(0.160)
Ln (the old age dependency ratio)	-0.415***	-0.635***	-0.723***	-0.616***	-0.663***
	(0.108)	(0.225)	(0.202)	(0.170)	(0.156)
Ln (the park green space area per capita)	-0.030	0.152	0.185	0.170	0.354***
	(0.099)	(0.106)	(0.114)	(0.122)	(0.135)
R-square	0.585	0.513	0.380	0.348	0.314
AIC	99.340	-248.387	-253.223	-256.775	-265.920
BIC	123.000	-221.348	-226.184	-209.457	-218.601
Log-likelihood	-42.670	132.194	134.612	142.388	146.960

^*^, ** and *** indicate significance at the level of 0.1, 0.05, 0.01, respectively. Rho, Spatial Autoregressive Parameters; Lambda, Residual lag parameter; Ln, natural logarithm; the SDM1 model is based on binary adjacency matrix, the SDM2 model is based on the geographical distance matrix. The value in parentheses represents the standard deviation

Table 8 shows the results of the spatial effect decomposition of the SDM models. Per capita GDP, social welfare expenditure as a percentage of GDP and the proportion of the tertiary industry had positive direct effects. For each 1% increase in these variables, the level of SECR increased by 0.537%, 0.135% and 0.913% respectively. The direct effect of population aging level on the allocation of SECR resources is negative. For every 1% increase in the old age dependency ratio, the level of SECR decreased by 0.561%. Population aging and service industry development have significant spatial spillover effects on the SECR allocation. For each 1% increase in the proportion of the tertiary industry in GDP and the old age dependency ratio in this region, the allocation level of SECR decreased by 0.800% and 0.528% in adjacent areas respectively. Apart from the variable “per capita park green space area”, the results of both SDM models were consistent, indicating the robustness of the SDM model.

**Table 8:** The results of the spatial effect decomposition of the SDM models

Variables	SDM1			SDM2		
	Direct effect	Indirect effect	Total effect	Direct effect	Indirect effect	Total effect
Ln (the per capita GDP)	0.537***	-0.131	0.406	0.404**	0.126	0.529
	(0.199)	(0.386)	(0.334)	(0.175)	(0.397)	(0.376)
Ln (the proportion of social welfare expenditure in GDP)	0.135**	0.186	0.321*	0.149**	0.275	0.424*
	(0.0679)	(0.154)	(0.194)	(0.0744)	(0.218)	(0.257)
Ln (the proportion of the tertiary industry in GDP)	0.913***	-0.800*	0.113	0.713***	-1.235**	-0.521
	(0.234)	(0.448)	(0.448)	(0.222)	(0.609)	(0.605)
Ln (the proportion of the older adults aged 65 +)	-0.242	-0.387	-0.629**	-0.261	-1.568***	-1.829***
	(0.155)	(0.296)	(0.299)	(0.160)	(0.382)	(0.411)
Ln (the old age dependency ratio)	-0.561***	-0.528**	-1.088***	-0.604***	-0.534	-1.138***
	(0.174)	(0.259)	(0.210)	(0.171)	(0.396)	(0.313)
Ln (the park green space area per capita)	0.153	0.225	0.378	0.249**	0.950	1.199**
	(0.111)	(0.348)	(0.382)	(0.117)	(0.604)	(0.609)

^*^, ** and *** indicate significance at the level of 0.1, 0.05, 0.01, respectively. the SDM1 model is based on binary adjacency matrix, the SDM2 model is based on the geographical distance matrix. The value in parentheses represents the standard deviation

## Discussion

### Evolution characteristics and the equity of SECR allocation

The findings of this study shows that the level of SECR in China is on the rise, but the overall level is not high. This suggests that proactive measures taken by the Chinese government to address the population aging, such as increasing input in elderly care resource, have yielded effective results. However, due to the large size of China's aging population, a substantial increase in per capita resource allocation in a short term is not feasible.

In addition, the allocation of SECR among the provincial-level administrative regions in China is also uneven. Compared to the central region, the eastern and western regions have relatively higher levels of SECR. One possible explanation is that the eastern region has a higher level of economic developmental and a relatively larger proportion of the tertiary industry, providing better financial and service support for the allocation of SECR. The western region lags behind the eastern and central regions in terms of economic development, but it has a relatively smaller size and proportion of elderly population than the other two regions.

There is a large gap in the fairness of SECR allocation between regions in China. The inequality of SECR allocation is higher in the eastern region compared to the western or central regions. The central region falls between the eastern and the western regions in terms of the comprehensive index of SECR, but it has a relatively lower Gini coefficient, indicating relatively fairer allocation of SECR in the central region.

The findings of this study reveal that the allocation of SECR in China is moving towards equalization, as evidenced by the overall decrease in the Gini coefficient. Since the regional gap mainly driven by inter-regional gap, it is important to focus on addressing the gaps between the regions, particularly between the eastern and the western regions, so as to improve the equity of the SECR allocation. The increase in the contribution rate of the super-variable density shows that the level of SECR allocation in the eastern region is not significantly ahead of other regions. Therefore, while focusing on addressing the regional gap, it is also important not to neglect the development of SEC in relatively less developed central regions.

### Spatial effects of SECR allocation

The findings of this study indicate the presence of spatial correlation in the allocation of SECR. This verifies the spatial correlation in the allocation of public welfare resources, which is consistent with the findings of the reference [55]. The supply of SECR exhibits spillover or free-riding effect. Regions with higher levels of SECR allocation may act as demonstration effects, influencing the surrounding areas to learn from and emulate their experiences and practices, resulting in higher SECR levels in the surrounding areas. However, regions with higher levels of SECR allocation may also generate competitive effect on the surrounding areas, attracting skilled labor force from the surrounding areas, which can hinder the improvement of SECR allocation level in the surrounding areas.

In addition, the local population aging has indirect negative spillover effects on the allocation of SECR in neighboring areas. The concentration of elderly population in specific areas stimulate the development of the elderly care service industry, which is conducive to attracting the labor force and social capital from the surrounding areas to participate in the construction of SECR. It may lead to the outflow of more advantageous resources in the surrounding areas, which hinders the improvement of SECR allocation in the surrounding areas.

### Policy implication for optimizing SECR allocation

The optimal allocation of SECR is proactive measures in respond to population aging, because resource allocation is a key foundation of the wellbeing and health of the older adults [29, 43, 45]. This study provides critical evidence for optimizing resource allocation by investigating the influencing factors of the SECR allocation from a spatial perspective. It contributes to understanding the path towards optimizing the SECR allocation, including enhancing the overall level of resource allocation and optimizing the resource allocation structure.

First, the attention should be given to the spatial adaptation of the SECR allocation. This study shows a significant spatial correlation in SECR allocation. Given the spillover or free-rider effects of public care supply, local governments consider their neighbors’ care supply decisions in their own decision-making processes [21, 38, 55]. The future trend of population aging is irreversible [17]. Our findings reveal a direct negative effect of population aging on the local regional allocation of SECR. As the old age dependency ratio increases locally, the challenges of providing elderly care become greater since the quantity of SECR cannot be rapidly increased within a short period. When the demand for SECR exceeds the supply, and too many old people occupy the limited SECR, it will result in a significant decline in the per capita availability of SECR and a lower level of regional allocation of elderly care resources. Therefore, SECR should be allocated based on the spatial distribution of the aging population. In areas with a concentration of elderly population, resource input should be increased accordingly to maintain the level of SECR capacity.

Second, the government should continue to increase the public financial input in elderly care resources. The findings of this study demonstrate that government’s social welfare expenditure has a positive impact on the allocation of SECR. Due to the intensification of aging population, it is necessary to increase the investment of elderly care resources to improve the level of per capita resources [4]. Considering that the public service nature of SECR, the government should increase public financial investment in elderly care resources, which may serve as a demonstration effect for the investment entities of various elderly care providers [44]. This will encourage enterprises, non-governmental organizations and other social capital to participate in the welfare provision of SECR.

Third, the elderly care service industry should be vigorously developed. This study shows that, when other factors were controlled, higher levels of development in the tertiary industry are associated with higher levels of SECR. The development of the tertiary industry not only promotes the expansion of the elderly care service industry but also stimulates the improvement in the service levels and quality [1], Ying, 2020; [52]. Digitalization and intelligent technologies will contribute to promoting the high-quality development of the pension industry, enhancing the output and efficiency of SECR utilization [23, 35, 38].

Fourth, the green ecological living environment should be improved to optimize the allocation of SECR. Although only the SDM2 model found a significant impact of per capita park green space area on SECR allocation, this finding reveals a potential correlation between the two. That is, the better the ecological environment, the higher the level of SECR allocation. Since older adults usually have more leisure time than younger people and prefer quiet and beautiful places, a good living environment provides basic elements of high-quality elderly care services. It helps attract investments from various stakeholders into elder care facilities [18].

### Limitations

We recognize at least two limitations of this study. First, this study only used provincial data to measure SECR which may ignore inter-provincial differences in SECR configuration. Although only inter-provincial differences can be studied, they are of value for a country with a large land size such as China. Second, the time span of the panel data used in this study is not long enough due to data availability constraints. Nonetheless, with a sample size of 217 in this study, it is reasonable to assume that the analysis is still reliable. Further research will continue to collect additional long-term data and explore the SECRs at the city-level to further improve the precision of the study.

## Conclusion

This study constructed a comprehensive index system to measure the level of SECR in China, and analyzes the spatiotemporal evolution characteristics and the influencing factors of SECR. The conclusions can be summarized as follows. First, the level of social elderly care resource in China is on an upward trend, but the overall level remains relatively low. Second, the fairness of the allocation of SECR has been improved, while the spatial distribution is imbalanced. Inter-regional differences are the main source of inequality. Third, economic development, fiscal input and service industry development have significant positive effects, while population aging has significant negative effects on the SECR allocation. Population aging and service industry development exhibit significant negative spatial spillover effects on the allocation of SECR. It is suggested that the country should maintain sustainable resource inputs, promote the development of the service sector and green ecological living environment, and decelerate the aging process of the population to achieve the optimal SECR allocation.

### Supplementary Information

**Additional file 1.**

## Data Availability

Publicly available datasets were analyzed in this study. This data can be found here: http://www.stats.gov.cn/tjsj/ndsj/, https://www.mca.gov.cn/article/sj/, https://www.yearbookchina.com/navibooklist-n3020013350-1.html. The datasets used and/or analyzed during the current study are also available from the corresponding author on reasonable request.
