# Supplementary material for: Spatiotemporal evolution and influencing factors of the allocation of social care resources for the older adults in China
Source: Int J Equity Health. 2023 Oct 18;22:222. doi: 10.1186/s12939-023-02007-0 (PMC10583468; doi:10.1186/s12939-023-02007-0)
Supplement: Supplementary file 1 — Additional file 1. [file 12939_2023_2007_MOESM1_ESM.docx]

Table S1 The comprehensive index of social elderly care resources in China (2013-2019)

| Provinces | 2013 | 2014 | 2015 | 2016 | 2017 | 2018 | 2019 | Mean | Ranking |
| --- | --- | --- | --- | --- | --- | --- | --- | --- | --- |
| Beijing | 0.615 | 0.585 | 0.586 | 0.695 | 0.744 | 0.664 | 0.578 | 0.638 | 1 |
| Shanghai | 0.499 | 0.587 | 0.520 | 0.570 | 0.522 | 0.494 | 0.454 | 0.521 | 2 |
| Jiangsu | 0.271 | 0.302 | 0.299 | 0.356 | 0.366 | 0.382 | 0.364 | 0.334 | 3 |
| Guangdong | 0.266 | 0.304 | 0.284 | 0.325 | 0.384 | 0.355 | 0.277 | 0.313 | 4 |
| Zhejiang | 0.288 | 0.340 | 0.279 | 0.318 | 0.340 | 0.326 | 0.280 | 0.310 | 5 |
| Qinghai | 0.222 | 0.208 | 0.225 | 0.283 | 0.326 | 0.378 | 0.402 | 0.292 | 6 |
| Guizhou | 0.198 | 0.296 | 0.363 | 0.320 | 0.334 | 0.261 | 0.197 | 0.281 | 7 |
| Xizang | 0.242 | 0.239 | 0.348 | 0.299 | 0.312 | 0.210 | 0.309 | 0.280 | 8 |
| Shaanxi | 0.216 | 0.213 | 0.220 | 0.247 | 0.287 | 0.225 | 0.214 | 0.232 | 9 |
| Tianjin | 0.240 | 0.218 | 0.214 | 0.243 | 0.262 | 0.248 | 0.189 | 0.231 | 10 |
| Sichuan | 0.114 | 0.183 | 0.204 | 0.312 | 0.286 | 0.246 | 0.202 | 0.221 | 11 |
| Xinjiang | 0.175 | 0.195 | 0.195 | 0.238 | 0.264 | 0.245 | 0.176 | 0.213 | 12 |
| Shandong | 0.236 | 0.219 | 0.196 | 0.207 | 0.243 | 0.155 | 0.138 | 0.199 | 13 |
| Inner Mongolia | 0.168 | 0.156 | 0.173 | 0.205 | 0.239 | 0.220 | 0.198 | 0.194 | 14 |
| Ningxia | 0.152 | 0.162 | 0.162 | 0.180 | 0.274 | 0.213 | 0.167 | 0.187 | 15 |
| Fujian | 0.141 | 0.145 | 0.181 | 0.212 | 0.229 | 0.203 | 0.197 | 0.187 | 16 |
| Hebei | 0.250 | 0.153 | 0.185 | 0.165 | 0.174 | 0.173 | 0.143 | 0.178 | 17 |
| Hubei | 0.146 | 0.158 | 0.169 | 0.197 | 0.184 | 0.200 | 0.188 | 0.177 | 18 |
| Liaoning | 0.173 | 0.179 | 0.155 | 0.179 | 0.189 | 0.168 | 0.123 | 0.167 | 19 |
| Heilongjiang | 0.150 | 0.127 | 0.207 | 0.179 | 0.117 | 0.188 | 0.133 | 0.157 | 20 |
| Anhui | 0.137 | 0.144 | 0.127 | 0.143 | 0.191 | 0.174 | 0.153 | 0.153 | 21 |
| Gansu | 0.154 | 0.145 | 0.141 | 0.161 | 0.134 | 0.179 | 0.144 | 0.151 | 22 |
| Shanxi | 0.137 | 0.154 | 0.132 | 0.170 | 0.173 | 0.140 | 0.121 | 0.147 | 23 |
| Hunan | 0.150 | 0.126 | 0.127 | 0.151 | 0.185 | 0.156 | 0.133 | 0.147 | 24 |
| Hainan | 0.124 | 0.124 | 0.112 | 0.148 | 0.178 | 0.181 | 0.115 | 0.140 | 25 |
| Chongqing | 0.116 | 0.104 | 0.112 | 0.156 | 0.166 | 0.161 | 0.153 | 0.138 | 26 |
| Yunnan | 0.113 | 0.121 | 0.120 | 0.130 | 0.147 | 0.123 | 0.119 | 0.125 | 27 |
| Jilin | 0.082 | 0.091 | 0.075 | 0.136 | 0.161 | 0.159 | 0.151 | 0.122 | 28 |
| Jiangxi | 0.130 | 0.119 | 0.096 | 0.121 | 0.111 | 0.129 | 0.140 | 0.121 | 29 |
| Guangxi | 0.121 | 0.061 | 0.097 | 0.127 | 0.128 | 0.151 | 0.139 | 0.118 | 30 |
| Henan | 0.084 | 0.101 | 0.096 | 0.092 | 0.103 | 0.105 | 0.152 | 0.105 | 31 |
| Eastern Region* | 0.282 | 0.287 | 0.274 | 0.311 | 0.330 | 0.304 | 0.260 | 0.293 | 1 |
| Central Region* | 0.127 | 0.127 | 0.129 | 0.149 | 0.153 | 0.156 | 0.146 | 0.141 | 3 |
| Western Region* | 0.166 | 0.174 | 0.197 | 0.222 | 0.241 | 0.218 | 0.202 | 0.203 | 2 |
| Mean | 0.197 | 0.202 | 0.206 | 0.234 | 0.250 | 0.233 | 0.208 | 0.219 | - |
| Coefficient of variation | 0.570 | 0.606 | 0.571 | 0.549 | 0.528 | 0.510 | 0.528 | 0.533 | - |

*According to the division of the three major economic zones by the National Bureau of Statistics of China, China is divided into eastern, central and western regions. 11 provinces are in the eastern regions, including Beijing, Tianjin, Hebei, Liaoning, Shanghai, Jiangsu, Zhejiang, Fujian, Shandong, Guangdong and Hainan; 8 provinces are in the central region, including Shanxi, Jilin, Heilongjiang, Anhui, Jiangxi, Henan, Hubei and Hunan; 12 provinces are in the western region, including Tibet, Inner Mongolia, Guangxi, Chongqing, Sichuan, Guizhou, Yunnan, Shaanxi, Gansu, Qinghai, Ningxia and Xinjiang.
